# The Peculiar Glycolytic Pathway in Hyperthermophylic Archaea: Understanding Its Whims by Experimentation In Silico

**DOI:** 10.3390/ijms18040876

**Published:** 2017-04-20

**Authors:** Yanfei Zhang, Theresa Kouril, Jacky L. Snoep, Bettina Siebers, Matteo Barberis, Hans V. Westerhoff

**Affiliations:** 1 Synthetic Systems Biology and Nuclear Organization, Swammerdam Institute for Life Sciences, University of Amsterdam, 1098 XH Amsterdam, The Netherlands; y.zhang2@uva.nl (Y.Z.); M.Barberis@uva.nl (M.B.); 2 Molecular Enzyme Technology and Biochemistry (MEB), Biofilm Centre, Centre for Water and Environment Research (CWE), University Duisburg—Essen, Universitätsstr. 5, 45141 Essen, Germany; theresak@sun.ac.za (T.K.); bettina.siebers@uni-due.de (B.S.); 3 Department of Biochemistry, University of Stellenbosch, Stellenbosch 7602, South Africa; j.l.snoep@vu.nl; 4 The Manchester Centre for Integrative Systems Biology, Manchester Institute for Biotechnology, School for Chemical Engineering and Analytical Science, University of Manchester, Manchester M1 7DN, UK; 5 Department of Molecular Cell Physiology, Vrije Universiteit Amsterdam, De Boelelaan 1085, 1081 HV Amsterdam, The Netherlands

**Keywords:** mathematical models, Archaea, flux, flux control coefficient, non-phosphorylating glyceraldehyde-3-phosphate dehydrogenase, GAPN

## Abstract

Mathematical models are key to systems biology where they typically describe the topology and dynamics of biological networks, listing biochemical entities and their relationships with one another. Some (hyper)thermophilic Archaea contain an enzyme, called non-phosphorylating glyceraldehyde-3-phosphate dehydrogenase (GAPN), which catalyzes the direct oxidation of glyceraldehyde-3-phosphate to 3-phosphoglycerate omitting adenosine 5′-triphosphate (ATP) formation by substrate-level-phosphorylation via phosphoglycerate kinase. In this study we formulate three hypotheses that could explain functionally why GAPN exists in these Archaea, and then construct and use mathematical models to test these three hypotheses. We used kinetic parameters of enzymes of *Sulfolobus solfataricus* (*S. solfataricus*) which is a thermo-acidophilic archaeon that grows optimally between 60 and 90 °C and between pH 2 and 4. For comparison, we used a model of *Saccharomyces cerevisiae* (*S. cerevisiae*), an organism that can live at moderate temperatures. We find that both the first hypothesis, i.e., that the glyceraldehyde-3-phosphate dehydrogenase (GAPDH) plus phosphoglycerate kinase (PGK) route (the alternative to GAPN) is thermodynamically too much uphill and the third hypothesis, i.e., that GAPDH plus PGK are required to carry the flux in the gluconeogenic direction, are correct. The second hypothesis, i.e., that the GAPDH plus PGK route delivers less than the 1 ATP per pyruvate that is delivered by the GAPN route, is only correct when GAPDH reaction has a high rate and 1,3-*bis*-phosphoglycerate (BPG) spontaneously degrades to 3PG at a high rate.

## 1. Introduction

Archaea, the third kingdom of life, have many unique properties that distinguish its members from most Bacteria and Eukarya. Many Archaea can grow in high temperature environments, like terrestrial hot springs, submarine hydrothermal, solfataric and abyssal hot vent systems [1]. The thermophilic Archaea thrive on temperatures between 60 and 80 °C, while hyperthermophiles live in the temperature range from 80 to 113 °C [2]. Temperature is a pervasive environmental factor that influences most biotic processes; unicellular organisms cannot shield themselves from it. Thus life at high temperature requires efficient adaption strategies. For example, the high temperatures always come with significant stress in terms of increased denaturation of proteins and membranes. When compared with the proteins from organisms that grow at moderate temperatures, proteins of hyperthermophiles are more stable at high temperatures and are often more resistant to chemical denaturants. The comparison of structural features from enzymes adapted to different temperature ranges revealed different adaptation strategies in (hyper)thermophilic proteins such as decrease in loop lengths, increase in oligomerization state and more distinct secondary structures [3]. Archaea have unique membrane spanning lipids with less hydrolysable ether linkages of glycerol to isoprenoid chains [4].

However, other problems associated with high or fluctuating temperatures cannot be resolved in this way, such as unfavorable equilibrium constants at high temperature of exothermic reactions and spontaneous decomposition of intermediates of metabolic pathways. One such example is found in the lower part of the Entner Doudoroff pathway, which can convert glyceraldehyde 3-phosphate (GAP) to pyruvate (Pyr). In the organisms that live at moderate temperatures this reaction is coupled to the phosphorylation of 2 molecules of adenosine 5′-diphosphate (ADP) to adenosine 5′-triphosphate (ATP) thereby storing a considerable part of the Gibbs free energy drop between GAP plus NAD^+^ and pyruvate plus NADH. The part of the pathway that is crucial for this example is the conversion of GAP plus phosphate to 1,3 bisphosphoglycerate (BPG) under reduction of NAD^+^ by GAPDH, followed by the phosphorylation of ADP by phosphoglycerate kinase (PGK)forming 3-phosphoglycerate (3PG). In terms of the standard Gibbs free energy, the former reaction is thermodynamically uphill and is aided by the latter, thermodynamically more downhill reaction (see also below). In the hyperthermophile *S. solfataricus* this mini-pathway may be bypassed by GAPN, which is a GAP dehydrogenase that yields 3PG directly without phosphorylating ADP [5]. The enzymes GAPDH, GAPN and PGK constitute a mini-network with a number of interesting network properties. Since the combination of GAPDH with PGK accomplishes the same as GAPN, save the production of ATP which is usually considered a good thing, it is not obvious why GAPN exists in this hyperthermophile organism and why it appears to be present in *S. solfataricus* and in all (hyper)thermophilic Archaea with optimal growth ≥80 °C studied so far [6].

An apparently attractive explanation is that the intermediate BPG is instable at high temperatures [7], and it might be degraded spontaneously into 3PG, that by bypassing BPG, GAPN prevents the loss of Gibbs energy by spontaneous hydrolysis of BPG to 3PG, still enabling harvest of ATP free energy at the pyruvate kinase step. However, this would still not clarify the functionality of GAPN: the normal GAPDH and PGK pathway should also yield this ATP even if all BPG decomposed spontaneously falling into 3PG. From this point of view, the GAPN pathway would never be better than the normal GAPDH plus PGK pathway. Consequently, our question is, if the above given reason does not suffice, why does GAPN exist in Archaea?

Because systems biology examines how biological function emerges from the nonlinear interactions between components in biological systems, and because biological networks tend to be fully connected, questions like this tend to meet with the curse of complexity [8]. Mathematical models can help to redress this complexity and to integrate experimental data, pre-existing knowledge and mathematical and biological theory. This can range from describing the topology of the networks and listing all their biochemical entities and their relationships with one another [9], to the discovery of biological functionality of compartmentation [10] or regulation [11]. The data required for the construction of mathematical models of biological systems can either be obtained in house [12] or taken from multiple literature sources [13]. Information about the metabolites and enzymes involved in a reaction can be found in databases such as BRENDA (BRaunschweig Enzyme Data, available online: http://www.brenda-enzymes.info/), KEGG (Kyoto Encyclopedia of Genes and Genomes, available online: http://www.genome.jp/kegg/) and Reactome (available online: http://www.reactome.org/), as well as in spreadsheet files that have been used to disseminate reconstructed models of metabolism from a number of organisms (available online: http://jjj.mib.ac.uk/). COPASI (Complex Pathway Simulator) can be used to construct mathematical models of biological systems and to calibrate parameters by fitting them to a set of experimental observations made from the biological system so that a more accurate response of the model can be attained in simulations [14]. In some cases, use can be made of pre-existing models in the live model repository JWS (Java Web Simulator)-online [15].

There are three main uses of precise mathematical modelling. The first one is to ensure consistency of the presumed understanding of a biological mechanism with existing data and physical chemical principles [16]. The second use is to predict behavior on the basis of biochemical knowledge that one assumes to be correct. The third one is much less known and perhaps even unappreciated: it helps to understand how biochemical networks may function, for example by developing and testing hypotheses in silico. It is a utility that one finds much in computational physics [17] but much less in biology, although examples have been forth coming [18]: if in some future the mathematical models of biological networks are essentially complete and if they have been validated sufficiently experimentally, they may take the position of the real networks by enabling the discovery of how these networks bring about their complex functions. At that point no further experimentation will be needed for discovery and validation of such network mechanisms; the process of validation may be confined to in silico studies.

In the present paper, we wish to demonstrate this third use of modelling of biochemistry. We shall assume that the information on the basis of which we build models is correct. We shall then use this third utility of modelling to try to increase the understanding of the metabolic organization of (hyper)thermophilic Archaea: we shall construct mathematical models and develop three hypotheses on the possible function of GAPN. We then test these hypotheses in silico and find that all three of them may have merit.

## 2. Results

### 2.1. The Scheme of the Model

**Figure 1:**
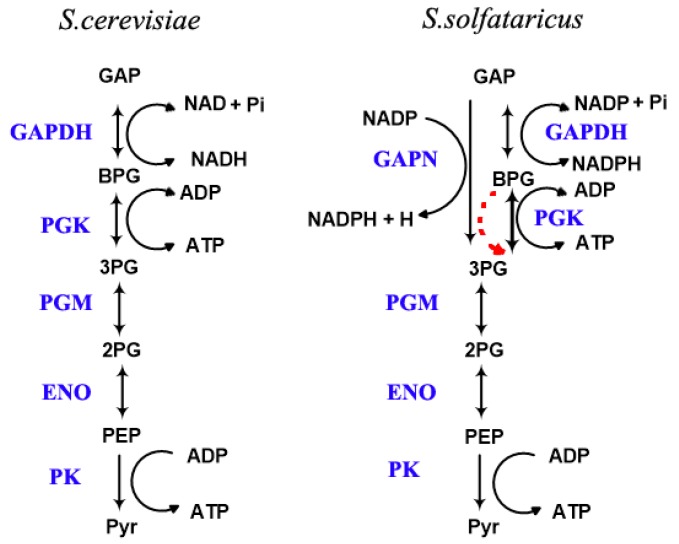
Schemes of the pathways converting glyceraldehyde 3-phosphate (GAP) to pyruvate, for *S. solfataricus* (on the right) and *S. cerevisiae* [19]. The dashed red arrow shows the degradation of 1,3 bisphosphoglycerate (BPG). The enzymes are showed in blue color. These pathways were modelled using a kinetic description for each reaction and integrating over time, using Copasi.

In this research we investigated the pathway from GAP to pyruvate of the hypothermophylic Archaea *S. solfataricus*. This pathway was compared with that in *S. cerevisiae* as *S. cerevisiae* is a typical model organism for unicellular Eukaryotes with optimal growth temperature of 30 °C (Figure 1). The reactions converting GAP to pyruvate are almost the same between the two organisms with only three exceptions. Apart from the normal GAPDH and PGK reaction, *S. solfataricus* also contains a GAPN reaction which can covert GAP to 3PG directly (Figure 1) using NADP^+^ as electron acceptor in the glyceraldehyde 3-phosphate dehydrogenase reaction. And another difference is that there is a non-enzymatic degradation of BPG in *S. solfataricus*: This degradation is temperature dependent and negligible in *S. cerevisiae* as its growth temperature is only 30 °C.

### 2.2. The Three Proposed Hypotheses

The pathway from GAP to pyruvate that runs through the GAPN reaction only produces 1 molecule ATP per pyruvate while the canonical GAPDH-PGK pathway to pyruvate can produce two molecules ATP. Here we propose three hypotheses that might explain the function of GAPN for the organism.

Our first hypothesis is that at high temperatures the GAPDH reaction becomes thermodynamically so unfavorable that it stalls. GAPDH gains a very high flux control coefficient (FCC), that the concentration of BPG gets very low and that the flux becomes very low. In this way GAPDH and PGK only cannot provide enough flux for the organism to survive.

At high temperatures BPG is not stable [7] and might be degraded spontaneously to 3PG without enzyme catalysis. The GAPDH and PGK route would then yield an ATP/pyruvate ratio smaller than 2 because the PGK reactions is bypassed by spontaneous hydrolysis of BPG to 3PG. The GAPN pathway would not have this problem, because it bypasses BPG and catalyzes the conversion of GAP directly to 3PG. However, the GAPN pathway has an ATP/Pyruvate flux ratio of 1 so that the expected flux ratio for the PGK + GAPDH pathway would always exceed 1. This in itself could therefore not explain the function of GAPN. Our second hypothesis is that in the PGK + GAPDH pathway the flux ratio of ATP produced over pyruvate produced could become less than 1, making that pathway inferior as compared to the GAPN pathway. We hypothesize that this could happen if the ATP produced at the pyruvate kinase level may be hydrolyzed by the reaction cycle constituted by PGK and BPG degradation: PGK is a reversible reaction and the increased conversion of BPG caused by spontaneous degradation at high temperatures might lead to an increase of the 3PG to BPG concentration ratio, thus enhancing the reverse reaction of PGK. In fact, even ATP generated in oxidative phosphorylation could be degraded in this way, potentially leading to a negative ATP/pyruvate flux ratio of the pathway between GAP and pyruvate.

The third hypothesis is that the three enzymes together enable the route between GAP and PEP to be traversed in both directions depending on the relative levels of PEP and GAP (and ATP/ADP). This would enable the organism to carry out gluconeogenesis hence to grow on C3-sugars as well as on C6 carbon sugars and thus increases metabolic flexibility.

### 2.3. In Silico Prevalidation of the Hypotheses

#### 2.3.1. Test of the First Hypothesis: Is the GAPDH Reaction Thermodynamically too Unfavorable?

The equilibrium constant (K_eq_) depends on the nature of reactants and products, the temperature, and the pressure. K_eq_ reflects the extent to which a chemical reaction can maximally be completed; if it exceeds 1 then the reaction can proceed for more than 50%, starting from 1 M concentrations of all reactants. Equation (4) (in Section 4.2.1) shows the thermodynamic origin of this: an equilibrium constant in excess of 1 then implies a drop in Gibbs energy across the reaction and hence that the reaction will operate in the forward direction until an excess of product over substrate has developed. We thus calculated the equilibrium constant (K_eq_) and the standard Gibbs free energy change (∆*G°*′) of the three most relevant reactions in our network at the two relevant temperatures (Table 1). The K_eq_ and ∆*G°*′ were defined in the catabolic direction. Results are shown in Table 1.

GAPDH is known as a thermodynamically unfavorable reaction, which will not proceed spontaneously readily. This would be indicated by a positive standard Gibbs energy change and an equilibrium constant smaller than 1. In cells this reaction is supposed to be aided by the subsequent PGK reaction which is a more thermodynamically favorable reaction, with a negative standard Gibbs energy change and an equilibrium constant much in excess of 1. The sum of the two reactions should then also have an overall net negative standard Gibbs free energy change and an overall equilibrium constant in excess of 1. This is the rationale for mesophiles such as the human and *S. cerevisiae*.

**Figure 2:**
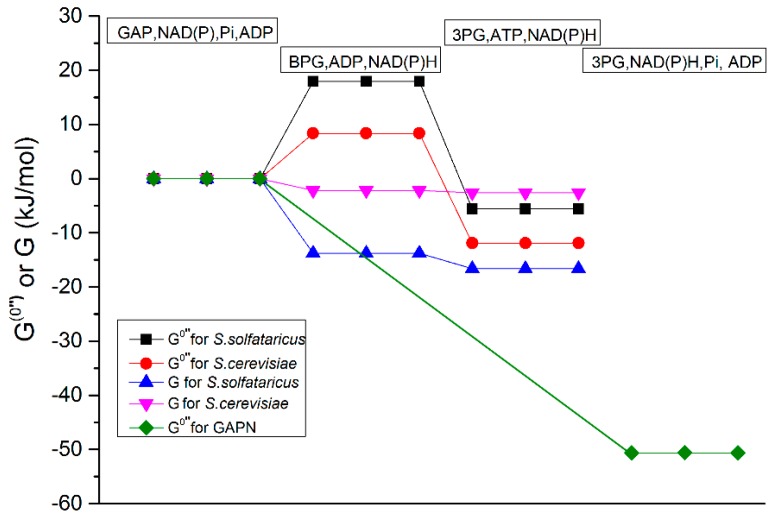
The Gibbs free energy changes and the steady state fluxes. The effective (∆*G*_0_′′ (*P*i = 10 mM)) standard Gibbs free energy change at 10 mM phosphate (black) of the GAPDH and PGK reactions at 70 °C, the effective standard Gibbs free energy change (red) at 30 °C in GAPDH and PGK reactions, the Gibbs energy change (blue) for *S. solfataricus* at 70 °C in GAPDH and PGK reactions, the Gibbs energy change (pink) for *S. cerevisiae* at 30 °C in GAPDH and PGK reactions, the effective standard Gibbs free energy change (green) for the non-phosphorylating glyceraldehyde-3-phosphate dehydrogenase (GAPN) reaction at 70 °C. The effective standard Gibbs free energy change is the one for a *P*i concentration of 10 mM (See Table 1). For the calculation of Gibbs energy change ∆*G*, we used for the substrates and products concentrations at the steady state calculated by the models (The models are in the folder named “hy1”. The supplementary word file named “Thermodynamics parameters” shows how we calculated all the thermodynamics parameters).

At 30 °C, which is most relevant for *S. cerevisiae*, the equilibrium constant of the GAPDH reaction is approximately 3.6 M^−1^, and the standard Gibbs energy for GAPDH at 30 °C is −3 kJ/mol, corresponding to a drop in Gibbs energy by 3 kJ/mol (Table 1). That this reaction is downhill in terms of standard Gibbs energy does not confirm the problem noted above. The standard Gibbs energy is defined for 1 M concentrations of all substrates and products. For realistic concentrations of reactants and products and because the number of reactants exceeds the number of products of GAPDH, the actual Gibbs energy drop across the reaction is much less than 3 kJ/mol and the reaction may even constitute a free energy ascent in order to accommodate this, we defined an “effective” standard Gibbs energy (G) comprising the phosphate concentration and this indeed showed that the GAPDH reaction is uphill at 30 °C, at least in terms of this effective Gibbs energy change for milliMolar concentrations of phosphate (Table 1 and Figure 2).

At 70 °C, which is relevant for *S. solfataricus*, this reaction is even less favorable, by a factor of approximately 20. The corresponding ∆*G°*′′ (at *P*i = 10 mM) of GADPH was even 18 kJ/mol at 70 °C, i.e., almost 40% of a Gibbs energy equivalent of ATP hydrolysis [20]. An inorganic phosphate concentration of 100 rather than 10 mM would alleviate this problem only by some 6.5 kJ/mol, bringing the effective equilibrium constant to 0.02, allowing 2% conversion at most.

The GAPDH reaction is aided by the subsequent PGK reaction. From Table 1, the K_eq_ of *S. cerevisiae* PGK is 3200 and the standard Gibbs free energy drop ∆*G°*′′ is −20 kJ/mol, while at 10 mM phosphate the GAPDH reaction only requires 8 kJ/mol effective Gibbs energy at 10 mM phosphate, so that the overall ∆*G°*′′ is −12 kJ/mol. The PGK reaction can pull the GAPDH reaction across the hill by lowering the concentration of BPG. Such pulling comes at a price however: the low BPG concentration would reduce the rate of the PGK reaction, but by substantial expression of that enzyme the flux through the step could still be high. This is how *S. cerevisiae* and humans appear to solve this thermodynamic issue leading to a drop of actual Gibbs energy across the two reactions combined (Figure 2). The actual magnitude of this drop also depends on the activity of the other enzymes down the metabolic pathway and should be established by dynamic models (in the folder named “hy1”).

In order to make the GAPDH reaction possible in the catabolic direction at high temperature (70 °C), the PGK reaction should be able to release more Gibbs energy that can be used to aid the GAPDH reaction, also at that temperature. From Table 1 we can see that at high temperatures K_eq_ of PGK is 3793, which is only slightly higher than that at low temperature (30 °C), and will release standard Gibbs free energy ∆*G°*′′ by some 24 kJ/mol. The overall ∆*G°*′′ of GAPDH and PGK if the inorganic phosphate concentration is 10 mM will then only be −6 kJ/mol, much smaller than the −12 kJ/mol at 30 °C. To pull the GAPDH reaction across its hill at 70 °C, it should reduce the BPG concentration by a factor of ten more. This would further reduce the PGK rate, but only a dynamic model comprising the steps further down the pathway may tell us that.

For the verdict on the hypothesis we implemented the integral kinetic models described in the Materials and Methods section. As shown in Table 2, the flux through GAPDH in *S. solfataricus* at 70 °C was only 0.14 mmol/min. Just as we predicted above, the BPG concentration at steady state in the *S. solfataricus* model was really low, i.e., only about 7 × 10^−6^ mM, while the K_M_ of PGK for BPG is 0.08 mM. In *S. cerevisiae* at 30 °C, the BPG concentration was about 0.007 mM, now above the K_M_ of PGK for BPG of 0.003 mM. This means that at high temperatures if *S. solfataricus* wants to increase the flux it needs to produce more BPG. This can also be justified by the high flux control coefficient of GAPDH in the *S. solfataricus* model, which means that the GAPDH reaction is the step that limits the flux most and that an increase in the activity of this reaction should increase the pathway’s steady state flux. In principle this could be achieved by an increased concentration of inorganic phosphate in the *S. solfataricus* cells (we calculated that an increase in phosphate from 10 to 100 mM would indeed increase the flux from 0.13 to 0.77 mM/min. The phosphate concentration in *S. solfataricus* cells is unknown however). For the *S. cerevisiae* model this enzyme’s flux control coefficient is only 0.1 (Table 2).

We next simulated the condition in which the *S. solfataricus* GAPDH would have a 1000 times increased enzyme activity through increasing its V_max_ in the model, in order to increase the BPG concentration so as to increase the flux. From Table 2 we can conclude that increasing GAPDH enzyme activity did increase the BPG concentration from 7 × 10^−6^ to 8 × 10^−4^ mM, and increased the flux from 0.14 to 7.4 mM/min.

We also simulated the conditions in *S. solfataricus* in which both GAPN and GAPDH/PGK exist. After we added GAPN to the model, the steady state GAP depletion flux became approximately 17 mM/min which is 120 times higher than in the condition with only GAPDH and PGK pathway.

As we discussed above if *S. solfataricus* increases the GAPDH concentration it can also increase its flux about 100 times. Accordingly, our question is why *S. solfataricus* should choose to use GAPN rather than to over express GAPDH? A first reason could be that over expressing GAPDH will require more mRNA, amino acids, tRNA and more Gibbs energy to make these, because it would need to be over expressed much more than GAPN is expressed. For rapidly growing cells this should not be very economic and it should be cheaper to express GAPN. A second reason could be that besides its glycolytic/gluconeogenesis function, GAPDH may also have some non-glycolytic functions “moonlighting enzyme”. It is reported that GAPDH is involved in cell death and DNA repair [21]. Although *S. solfataricus* is different from eukaryotes, its GAPDH might also have some non-glycolytic functions.

Our results showed that having only GAPDH and PGK is not enough for supporting *catabolism* through the bottom glycolytic pathway in *S. solfataricus* at a substantial rate. And the strain needs GAPN to provide more flux in order to survive, or should overexpress GAPDH to an extent that would be more costly than the expression of GAPN. Although for our model of *S. solfataricus* we showed that GAPN should be essential for glycolysis at a sufficient flux, this should not be taken as consequential for this organism in actual practice. Our model is for the low glycolytic pathway only and foregoes the existences and possible activity of the KDG pathway that bypasses this pathway. Indeed, upon deletion of KDG kinase, which should shut down the pathway that we model, *S. solfataricus* still grows on glucose [22]; the non-phosphorylative branch may take over, as oxidative phosphorylation of pyruvate can deliver much ATP. Our findings may be more relevant for an organism such as *Thermococcus kodakarensis* where GAPN is glycolytically essential [23]. We emphasize that this paper is meant to show what can be achieved in terms of in silico discovery using dynamic models. This paper does not focus on the actual discoveries that we and others are making with this method.

We conclude that the first of our three hypotheses is plausible in silico. From Table 2 we can see that even with GAPN, GAPDH and PGK in the model, the flux of *S. solfataricus* model was still 13 times lower than that of *S. cerevisiae* model*.* On glucose, *S. cerevisiae* has a doubling time of around 100 min at 30 °C [24], While *S. solfataricus* has a doubling time of 20 h at 70 °C [25], which gives us a 12-fold difference. And this difference is consistent with our model findings. Yet, the absolute specific growth rates predicted for either organism deviates from the experimental values. We point out that our models are still far from having been validated completely.

#### 2.3.2. Test of the Second Hypothesis: Would There Be an Excessive ATP Loss?

**Figure 3:**
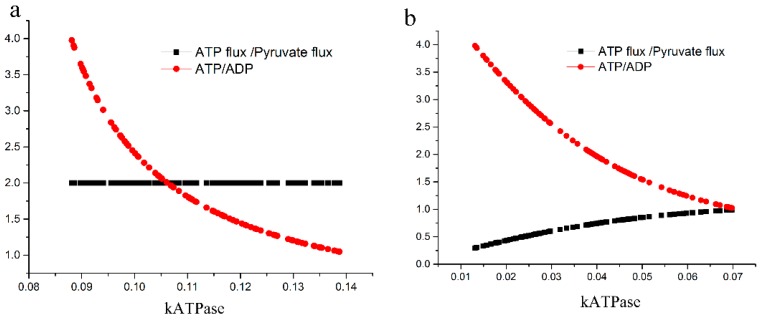
The adenosine 5′-triphosphate (ATP) production flux to the pyruvate production flux at various BPG decomposition rates for *S. solfataricus*. (**a**) Low (realistic) and (**b**) high rate constants of BPG decomposition were set by taking *k*_deg_ equal to 1.058 and 105,800 min^−1^ respectively.

Our second hypothesis is that in the BPG + GAPDH pathway the ratio of the ATP production flux to the pyruvate production flux could become less than 1, making that pathway inferior as compared to the GAPN pathway. Differently from when we were testing the first hypothesis (see above), we added an ATP hydrolysis (ATPase) reaction to the model and set ATP and ADP as variables with a constant sum concentration. As the ratio of ATP/ADP is expected to reside between 1 to 4 physiologically, we executed a parameter scan for the ATPase rate constant: we determined all the possible ATPase rate constants that could give an ATP/ADP ratio between 1 and 4 (Figure 3).

Kouril and her colleagues [7] assayed the non-enzymatic degradation of BPG at 70 °C. Its first order *k*_deg_ was approximately 1.06 min^−1^. When we used this *k* value in our model, the ratio of ATP flux to PYK flux was almost 2 (Figure 3a); if this *k* value is around 1 min^−1^ the spontaneous degradation of BPG should have little influence on the ATP yield of the pathway. Figure 3b serves as the positive control for the methodology: when we increased *k*_deg_ to a very high value like 106,000, the ATP flux to PYK flux did become lower than 1; the higher the ATP concentration the lower the ratio of ATP flux to Pyruvate flux. We conclude that our second hypothesis could be right only if the BPG spontaneous decomposition were much higher than what has been observed at 70 °C.

In these calculations, the fluxes through the lower half of the glycolytic pathway were low, e.g., only 0.14 mM/min. This is due the thermodynamic problem noted above when analyzing the first hypothesis within GAPDH and PGK reactions. *S. solfataricus* might overcome this problem by accumulating GAP and NADP^+^, and allowing NADPH to be lowered by an additional oxidation reaction. In addition, the intracellular phosphate concentrations could be as high as 100 mM. When we made the GAPDH reactions thermodynamically more favorable by improving its substrate/product ratios, we did indeed observe an increase in flux through PYK from 0.136 mM/min to an average flux of 20 mM/min.

**Figure 4:**
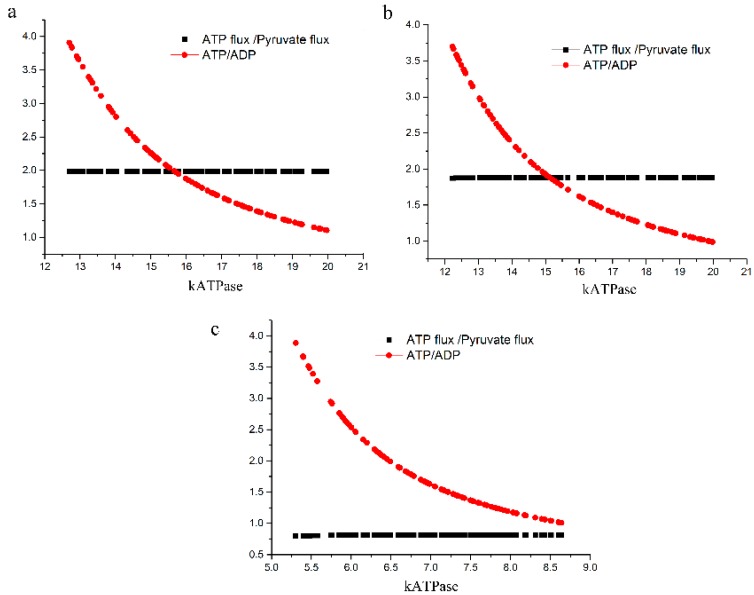
The ratio of the ATP consumption flux to the pyruvate production flux at various BPG decomposition rates for *S. solfataricus* in the models with more reasonable fluxes towards pyruvate. (**a**) *k*_deg_ was set to 1.058 min^−1^; (**b**) *k*_deg_ was set to 10.58 min^−1^; (**c**) *k*_deg_ was set to 105.8 min^−1^.

The growth temperatures of *S. solfataricus* are between 70 and 90 °C. The BPG decomposition rate constant at 70 °C is 1.058 min^−1^ [7]. When in our model with more reasonable fluxes towards pyruvate, we assumed that the degradation rate constant at 80 °C is 10 times of that at 70 °C (corresponding to a temperature coefficient Q_10_ of as much of 10 where 3 is more normal), the ATP flux/pyruvate production ratio dropped from 1.99 (Figure 4a) to around 1.87 (Figure 4b). We also examined the effect of assuming that the degradation rate constant at 90 °C is 100 times of that at 70 °C and implemented this condition in our model. Now the ATP/pyruvate production ratio dropped to around 0.81 (Figure 4c). In an earlier modelling study, Kouril et al. [19] reported that at high temperatures ATP/Pyr production ratio dropped to 0.8, which is in good agreement with our finding here.

From this point of view, our second hypothesis could become realistic at very high temperatures or if conditions such as bivalent ion concentrations in *S. solfataricus* should much favor its spontaneous hydrolysis.

#### 2.3.3. Test of the Third Hypothesis: Is Traffic Bidirectional?

The third hypothesis is that the three enzymes together in *S. solfataricus* enable the route between GAP and PEP to be traversed in both directions depending on the relative levels of PEP and GAP (and on the ATP/ADP ratio) and thereby bring the functionality to the organism of being able to carry out both gluconeogenesis and the lower half of glycolysis.

Table 3 shows our results. Under conditions showing in the caption of Table 3, the *S. solfataricus* in silico pathway would carry out glycolysis. In *S. cerevisiae*, its fluxes were all the same, while in *S. solfataricus*, the flux through GAPDH and PGK were much lower than that of the flux through GAPN and even in the opposite direction.

Table 4 shows that under the conditions (low NAD(P)/NAD(P)H ratio and high concentration of PEP and low concentration of GAP) showing in the caption of Table 4, the *S. solfataricus* GAPDH-PGK pathway would carry out gluconeogenesis as witnessed by all fluxes except that through GAPN becoming negative. In *S. cerevisiae*, the corresponding fluxes were all below zero.

Therefore, we reach the conclusion that in *S. solfataricus* GAPDH and PGK may mainly function under gluconeogenic conditions and GAPN is responsible for glycolysis, whereas in *S. cerevisiae* GAPDH and PGK could well function under both conditions in the appropriate direction.

## 3. Discussion

In this paper, we formulated three hypotheses that might have explained why GAPN is essential in hyperthermophilic organisms. Kinetic models are based on the kinetic parameters measured experimentally, so the accuracy is depending on the accuracy of the experiment data. In our model we used some parameters of *S. cerevisiae* for *S. solfataricus* models, which may be different from the ones for *S. solfataricus*. However, for the important parameters like the ones for PGK, GAPDH and GAPN we used the ones measured for *S. solfataricus.* Here we just demonstrated the use of kinetic models to help us to test hypotheses, i.e., not for quantitative but for qualitative purposes.

We also constructed corresponding models of yeast as a control validating that yeast should not require GAPN. We found that GAPN may function to enable glycolytic flux and the combination of GAPDH and PGK may enable gluconeogenic flux. The hypothesis that GAPN exists to prevent a drop of the ratio of ATP synthesis flux to pyruvate flux below the value of 1 was not upheld for the known decomposition rate of BPG at 70 °C. Only if that decomposition was much higher at temperatures around 90 °C for instance, this hypothesis might still be valid.

An overarching aim of this paper was to demonstrate the research strategy of in silico discovery for Biology: assuming that the kinetic and thermodynamic parameters we used were right, we have here discovered more about the function of GAPN in hyperthermophilic organisms. We found that it enables the organism to carry out the lower half of glycolysis at high temperatures, which would otherwise be impossible because the GAPDH reaction is thermodynamically too much uphill with the PGK reaction not delivering enough Gibbs energy drop to pull the GAPDH reaction through by lowering the BPG concentration. We also discovered in silico that the reasons that GAPN exists is not because the ATP/pyruvate ratio of the GAPDH + PGK pathway would become lower than that of the GAPN pathway, unless at much higher temperature the BPG should be much less stable than at 70 °C. With more and more systems biology models becoming available, the use of in silico methods for the discovery of network mechanisms that serve important biological functions should become even more productive. This is of course not limited to Archaea. Also hyperthermophilic Bacteria will be of great interest. For instance, *Thermotoga neapolitiana* is already being modeled [26].

None of our conclusions other than the one that in silico discovery has become possible also in biology [15], should be taken as definitive. Intracellular concentrations and activities may not be quite what we think they are and so may be intracellular enzyme activities [27]. Metabolism may be channeled [28] and more dynamic [29] or crowded [30] than we here considered it to be. But confrontation of the results of in silico discovery with precise experimental biology may get us where we want [31] or need [32] to be.

## 4. Materials and Methods

### 4.1. Model Construction

The reactions modelled for *S. cerevisiae* and *S. solfataricus* were the same except that apart from the GAPDH and PGK reaction *S. solfataricus* also contained a bypass reaction GAPN. As BPG may decompose spontaneously to 3PG, to the *S. solfataricus* models the non-enzyme catalyzed conversion of BPG to 3PG was added. To obtain our “*S. solfataricus*” models we refer to the method used by Kouril et al. [19]. For the *S. cerevisiae* model rather than to use the one published by Teusink et al. [22] in 2000 we used the more recently published by Smallbone et al. [12] deleting the reactions that are not in Figure 1. As the latter model did not include inorganic phosphate (*P*i) in the GAPDH reaction explicitly, we changed the reaction of GAPDH from “GAP + NAD ↔ BPG + NADH” to “GAP + NAD + *P*i ↔ BPG + NADH”. And we also changed its rate law (Table 5). Moreover, instead of the V_max_’s used in the model, we used the V_max_ of the enzymes measured under in vivo medium conditions in *S. cerevisiae* cell extract [33]. Apart from changing the reactions of GAPDH and GAPN of the 2012 model [19], we also changed the PGK reaction using the kinetics published by Kouril [34,35]. As we want to investigate why GAPN exists in *S. solfataricus*, it should ultimately be more reasonable for us to change all the parameters and rate laws for all of these three enzymes, but because this information for *S. solfataricus* is still unknown and because this paper is about principal use of the modelling approach in in silico hypothesis checking than about ultimate conclusions, the parameters for GPM, ENO, PYK reactions were kept identical to the ones in the *S. cerevisiae* model except for their V_max_’s (For the V_max_ we used those of the *S. solfataricus* cell extract published in [34]). Initial concentrations of boundary metabolites have been fixed to in vivo concentrations in *S. cerevisiae* [33]. All rate equations and parameters are shown in Table 5. The model files can be found in supplementary materials and will be available from JWS-online (available online: https://jjj.bio.vu.nl/) upon acceptance for publication. We used Copasi (COPASI 4.16, available online: http://copasi.org/News/2015/08/19/Release/) throughout for the modelling and this software has been included in the supplementary files in the folder named copasi.

### 4.2. Hypotheses Tests and Simulations

#### 4.2.1. Test of the First Hypothesis; Thermodynamically Unfavorable GAPDH

For the *S. cerevisiae* model, after setting all the initial concentrations we ran the steady state, time course and metabolic control analysis options in Copasi in order to obtain the species concentrations, the flux of each reaction and the flux control coefficients. This model has been included in the supplementary files in the folder named “hy1” and the name of the model is “*S. cerevisiae* hy1”.

For *S. solfataricus* we first ran the model with the reaction rate of GAPN at 0 (The name of this model is “*S. solfataricus* hy1” in the folder named as hy1). And then we ran the model with V_max_ of GADPH increased 1000 times in the model (The name of this model is “*S. solfataricus* hy1 increased GAPDH V_max_” stored in the folder named hy1). Finally, we tested the situation when the GAPN reaction had been added to the model (The name of this model is “*S. solfataricus* hy1 with GAPN added”, stored in the folder named hy1). Running “Steady-State” of the models for first hypothesis can get all the data.

The equilibrium constants are defined as the ratio of all product concentrations multiplied to all substrate concentrations multiplied, all in M units. For the calculation of the equilibrium constant (K_eq_) for PGK in *S. solfataricus*, we used the Briggs-Haldane relation (Formula (1)) obtained by equating the forward to the reverse rate of the reaction:

(1) $${\mathrm{Keq}}_{\mathrm{PGK}}=\frac{\left[V_{mfPGK}][K_{PGKATP}][K_{PGK3PG}\right]}{\left[V_{\mathrm{mr}PGK}][K_{PGKADP}][K_{PGKBPG}\right]}=\frac{\frac{37.96U}{mg}\times 9.309mM\times 0.567mM}{\frac{17.212U}{mg}\times 0.374mM\times 0.0082mM}=3793$$

For the calculation of K_eq_ for GAPDH in *S. solfataricus*, we again used the Briggs-Haldane relation:

(2) $${\mathrm{Keq}}_{\mathrm{GAPDH}}=\frac{\left[V_{mfGAPDH}][K_{GAPDHNADPH}][K_{GAPDHBPG}\right]}{\left[V_{\mathrm{mr}GAPDH}][K_{GAPDHNADP}][K_{GAPDHGAP}][K_{GAPDHPi}\right]}=\frac{\frac{35.551U}{mg}\times 0.09445mM\times 0.0895mM}{\frac{23.6115U}{mg}\times 0.204mM\times 3.1091mM\times 108.32mM}=0.18M^{-1}$$

For K_eq_ of PGK in *S. cerevisiae* we used the one in the model, which had the value of 3200. While for K_eq_ of GAPDH in *S. cerevisiae*, we calculated it according to the following Formula (3).

(3) $$\mathrm{Keq}=\frac{\mathrm{keq}}{K_{Pi}}=\frac{0.0054}{0.0015M}=3.6M^{-1}$$

Here k_eq_ = 0.0054 is the value in the original model [22], while *K_Pi_* is 0.0015 M [36]. In order to investigate the influence of phosphate for GAPDH, we named the standard Gibbs free energy ∆*G*°′ and calculated it using the following Formula (4).

(4) $$\Delta G^{0\prime }=-RT\ln\left(\mathrm{Keq}\right)$$

*R* is the gas constant of 8.314 J·K^−1^·mol^−1^. *T* is temperature in Kelvin (unit K). For calculation of the ∆*G°*′ for *S. solfataricus* the *T* value is 347 K; for that of the ∆*G*′′ of *S. cerevisiae* the *T* value was taken to be 307 K. The effective standard Gibbs energy change, which takes the influence of the phosphate being much lower than the canonical 1 M into account, was named ∆*G*°′′ for GAPDH. We used the following formula (Formula (5)) to calculate ∆*G°*′′.

(5) $$\Delta G^{0\prime \prime }=\Delta G^{0\prime }+RT\ln\frac{1}{Pi}$$

Here *P*i was 1, 10, or 100 mM as indicated. As phosphate has no influence on the standard Gibbs energy change for PGK, we used the following formula to calculate ∆*G°*′′ for PGK

(6) $$\Delta G^{0\prime \prime }=-RT\ln\left(\mathrm{Keq}\right)$$

For the calculation of ∆*G*, we used the following formula (Formula (7)).

(7) $$\Delta G=\Delta G^{0\prime \prime }+RT\ln\frac{\left[P_{1}][P_{2}\right]}{\left[S_{1}][S_{2}\right]}$$

[*P*_1_] and [*P*_2_] represent the product concentrations at steady state and [*S*_1_] and [*S*_2_] represent substrate concentrations at steady state. We calculated the Gibbs free energy change, for the substrates and products using the steady state substrates and products concentration values calculated by the models (The models are in the folder named “hy1”). The supplementary word file named “Thermodynamics parameters” showed how we calculated all the thermodynamics parameters.

#### 4.2.2. Test of the Second Hypothesis; Excessive Loss of ATP

As our second hypothesis is that in the PGK + GAPDH pathway the flux ratio of ATP produced over pyruvate produced could become less than 1, we couldn’t fix ATP and ADP. So we added an ATPase reaction in the models for testing the second hypothesis. Since in living cells the ATP concentration is normally in between 1 and 4, we did a parameter scan for the ATPase rate constant in order to find all the possible conditions that were congruent with this. The first we tested was the condition that the k value for decomposition of BPG was 1.058 min^−1^ [7] (The name of this model is “*S. solfataricus* hy2 K = 1.058 ATP and ADP ratio ranging from 1 to 4”). The second we tested was the condition that the k value was 100,000 times larger (The name of this second model is “*S. solfataricus* hy2 K = 105,800 ATP and ADP ratio ranging from 1 to 4”).

In the above calculations, the fluxes through the lower half of the glycolytic pathway were low, which was due to the thermodynamic problem to be noted when analyzing the first hypothesis. Therefore, we also simulated conditions in which *S. solfataricus* should overcome this problem by increasing GAPDH enzyme activity 10 times (through increasing the V_max_ of GAPDH 10 times), by increasing the substrates GAP, *P*i concentrations each 10 times, and by lowering NADPH to 0.1 mM and also increasing the NADP to 1.59 mM (The name of this model is “*S. solfataricus* hy2 K = 1.058 increased substrate concentration ATP and ADP ratio ranging from 1 to 4”).

The growth temperatures of *S. solfataricus* are between 70 and 90 °C. The degradation rate constant for BPG at 70 °C is 1.058 min^−1^ [7]. We assumed that the degradation rate constant at 80 °C was 10 times of that at 70 and 90 °C was 100 times of that at 70 °C. Then we simulated these conditions in the model with ATP/ADP ratio ranging from 1 to 4 by changing the *k*_degradation_ from 1.058 to 10.58 min^−1^ (“*S. solfataricus* hy2 K = 10.58 increased substrates concentrations ATP and ADP ratio ranging from 1 to 4”) and from 1.058 to 105.8 min^−1^ (“*S. solfataricus* hy2 K = 105.8 increased substrates concentrations ATP and ADP ratio ranging from 1 to 4”).

Running “Parameter Scan” of the models for third hypothesis can get all the data.

#### 4.2.3. Test of the Third Hypothesis; Bidirectional Traffic through GAPDH Plus PGK and GAPDH

We first simulated the production of PEP from GAP for *S. cerevisiae* and *S. solfataricus*. The initial concentrations of the species were fixed as follows: GAP 0.15 mM, NAD (NADP) 1.2 mM, NADH (NADPH) 0.39 mM, ADP 1.32 mM and ATP 2.52 mM. For all other metabolic intermediates, the initial concentrations were 0 mM and these were allowed to evolve in the simulations. Then we ran the time course and obtained the flux of each enzyme at steady state.

Next we simulated the production of GAP from PEP. In this condition the initial concentrations of species were changed. The initial species concentrations were set as follows: GAP 0.015 mM, NAD (NADP) 0.1 mM, NADH (NADPH) 1.49 mM, PEP 0.7 mM, ADP 1.32 mM and ATP 2.52 mM. For the other metabolic intermediates, the initial concentrations were 0 mM and again allowed to develop in the simulations. We ran the time course and obtained the steady state flux through each enzyme. The models are in the folder named “hy3”. Running “Steady-State” of the models for third hypothesis can get all the data.

## 5. Conclusions

In this paper, we formulated three hypotheses that might have explained why GAPN is essential in hyperthermophilic organisms. We demonstrated the use of kinetic models to help us to test hypotheses, i.e., not for quantitative but for qualitative purposes. After simulating we found that GAPN may function to enable glycolytic flux and that the combination of GAPDH and PGK may enable gluconeogenic flux. The hypothesis that GAPN exists to prevent a drop of the ratio of ATP synthesis flux to pyruvate flux below the value of 1 was not upheld for the known decomposition rate of BPG at 70 °C.

## Figures and Tables

**Table 1:** Equilibrium constants and standard Gibbs free energy changes of glyceraldehyde-3-phosphate dehydrogenase (GAPDH) and phosphoglycerate kinase (PGK) at 30 and 70 °C. For the PGK reaction the effective standard Gibbs energy increase ∆*G*°′′ is defined as [−*RT*ln(K_eq_)], for GAPDH reaction ∆*G*°′′ is defined as per −*RT*ln(K_eq_) + *RT*ln(1/*P*i) where the K_eq_ is the concentration ratio at equilibrium of products to substrates and *P*i the concentration of inorganic phosphate indicated in the first column.

Name	GAPDH	PGK	GAPDH	PGK
Temperature	70 °C	70 °C	30 °C	30 °C
K_eq_	0.18 M^−1^	3793	3.6 M^−1^	3200
∆*G*°′′ (*P*i = 1 mM)	24.4 kJ/mol	−23.5 kJ/mol	14.2 kJ/mol	−20.3 kJ/mol
∆*G*°′′ (*P*i = 10 mM)	17.9 kJ/mol	−23.5 kJ/mol	8.4 kJ/mol	−20.3 kJ/mol
∆*G*°′′ (*P*i = 100 mM)	11.4 kJ/mol	−23.5 kJ/mol	2.6 kJ/mol	−20.3 kJ/mol
∆*G*°′′ (*P*i = 1 M) ≡ ∆*G*_0_′	4.9 kJ/mol	−23.5 kJ/mol	−3.2 kJ/mol	−20.3 kJ/mol

**Table 2:** Modelled fluxes (mM/min), concentrations (mM) and flux control coefficients at steady state for *S. cerevisiae* (at 30 °C) and for *S. solfataricus* (at 70 °C). Pathways were as in Figure 1. C^J^: flux control coefficients of the 3PG production flux with respect to the enzyme indicated.

Reactions between GAP and 3PG Included	*S. cerevisiae* (30 °C)	*S. solfataricus* (70 °C)		
	PGK, GAPDH	PGK, GAPDH	PGK and 1000 Times GAPDH	PGK, GAPDH, GAPN
J_GAPDH_	236	0.136	7.4	−0.232
J_PGK_	236	0.136	7.4	−0.234
J_GAPN_	---	---	---	16.82
BPG	0.007	6.9 × 10^−6^	8.1 × 10^−4^	2.4 × 10^−3^
C^J^_GAPDH_	0.11	0.99	0.03	−0.011
C^J^_PGK_	0.01	0.005	0.40	0

The symbol “---” represents that GAPN does not exist in the model.

**Table 3:** The fluxes from glyceraldehyde 3-phosphate (GAP) to phosphoenolpyruvic acid (PEP) under catabolic conditions. Initial concentrations of the species were fixed as follows: GAP 0.15 mM, NAD (NADP) 1.2 mM, NADH (NADPH) 0.39 mM, ADP 1.32 mM and ATP 2.52 mM. And for other metabolic intermediates the initial concentrations were 0 mM and evolved in the simulations.

Flux (mM/min)	GAPDH	GAPN	PGK	ENO	PGM
*S. cerevisiae*	235	---	235	235	235
*S. solfataricus*	−0.23	16.82	−0.23	16.59	16.59

The symbol “---” represents that GAPN does not exist in the model.

**Table 4:** The fluxes from PEP to GAP under anabolic conditions. The initial species concentrations were fixed as follows: GAP 0.0015 mM, NAD (NADP) 0.1 mM, NADH (NADPH) 1.49 mM, PEP 0.7 mM, ADP 0.1 mM and ATP 3.74 mM. For other metabolic intermediates the initial concentrations were 0 mM and allowed to evolve in the simulations.

Flux (mM/min)	GAPDH	GAPN	PGK	ENO	PGM
*S. cerevisiae*	−107	---	−107	−107	−107
*S. solfataricus*	−1.6	0.7	−1.6	−0.83	−0.83

The symbol “---” represents that GAPN does not exist in the model.

**Table 5:** The enzymes, rate laws and parameters used in the models.

Enzymes	Rate Laws	Parameters
GAPDH for *S.cerevisiae*	$\;\frac{V_{mGAPDH}\times \left(\frac{GAP\times NAD\times Pii}{K_{mGAPDHGAP}\times K_{mGAPDHNAD}\times K_{mGAPDHPi}}-\frac{BPG\times NADH}{\left(K_{eqGAPDH}\times K_{mGAPDHGAP}\times K_{mGAPDHNAD}\right)}\right)}{\left(\left(1+\frac{GAP}{K_{mGAPDHGAP}}\right)\times \left(1+\frac{Pii}{K_{mGAPDHPi}}\right)+\frac{BPG}{K_{mGAPDHBPG}}\right)\times \left(1+\frac{NAD}{K_{mGAPDHNAD}}+\frac{NADH}{K_{mGAPDHNADH}}\right)}$	*V_mGAPDH_* = 1859 mM/min [33]
		*K_GAPDHGAP_* = 0.21 mM [12]
		*K_eqGAPDH_* = 0.0054 [12]
		*K_GAPDHNAD_* = 0. 09 mM [12]
		*K_GAPDHNADH_* = 0.06 mM [12]
		*K_GAPDHBPG_* = 0.0098 mM [12]
		*K_GAPDHPi_* = 1.5 mM [12]
PGK for *S.cerevisiae*	$\frac{V_{m\mathrm{rPGK}}\times \left(\frac{-ATP\times 3PG}{K_{mPGKATP}\times K_{mPGK3PG}}+\frac{K_{\mathrm{eq}PGK}\times ADP\times BPG}{K_{mPGKATP}\times K_{mPGK3PG}}\right)}{\left(1+\frac{ADP}{K_{iADP}}\right)\times \left(1+\frac{3PG}{K_{mPGK3PG}}\times \left(1+\frac{ATP}{K_{mPGKATP}}\right)+\frac{BPG}{K_{mPGKBPG}}+\left(1+\frac{ADP}{K_{mPGKADP}}\right)\right)}$ ^1^	*V_mrPGK_* = 2670 mM/min [33]
		*K_PGKADP_* = 0.2 mM [12]
		*K_PGKATP_* = 0.3 mM [12]
		*K_PGKBPG_* = 0.003 mM [12]
		*K_PGK3PG_* = 0.53 mM [12]
		*K_eqPGK_* = 3200 [12]
PGMA	$\frac{\frac{V_{mPGMA}}{K_{mPGMA3PG}}\times \left(3PG-\frac{2PG}{K_{\mathrm{eq}PGMA}}\right)}{1+\frac{3PG}{K_{mPGMA3PG}}+\frac{2PG}{K_{mPGMA2PG}}}$	*V_mPGMA for S.cerevisiae_* = 856 mM/min [33]
		*V_mPGMA for S. solfataricus_* = 56 mM/min [34]
		*K_mPGMA2PG_* = 0.08 mM [12]
		*K_mPGMA3PG_* = 1.2 mM [12]
		*K_eqPGMA_* = 0.19 mM [12]
ENO	$\frac{\frac{V_{mENO}}{K_{mENO2PG}}\times \left(2PG-\frac{PEP}{K_{eqENO}}\right)}{1+\frac{2PG}{K_{mENO2PG}}+\frac{PEP}{K_{mENOPEP}}}$	*V_mENO for S.cerevisiae_* = 357 mM/min [33]
		*V_mENO for S. solfataricus_* = 20.5 mM/min ^2^
		*K_mENO2PG_* = 0.04 mM [12]
		*K_mENOPEP_* = 0.5 mM [12]
		*K_eqENO_* = 6.7 mM [12]
PYK	$\frac{V_{mPYK}\times \left(\frac{ADP\times PEP}{K_{mPYKADP}\times K_{mPYKPEPE}}-\frac{ATP\times PYR}{K_{mPYKADP}\times K_{eqPYK}\times K_{mPYKPEPE}}\right)}{\left(1+\frac{ADP}{K_{mPYKADP}}+\frac{ATP}{K_{mPYKATP}}\right)\times \left(1+\frac{PEP}{K_{mPYKPEPE}}+\frac{PYR}{K_{mPYKPYR}}\right)}$	*V_mPYK for S.cerevisiae_* = 559 mM/min [34]
		*V_mPYK for S. solfataricus_* = 76 mM/min [34]
		*K_mPYKADP_* = 0.53 mM [12]
		*K_mPYKATP_* = 1.5 mM [12]
		*K_mPYKPEP_* = 0.14 mM [12]
		*K_mPYKPYR_* = 21 mM [12]
		*K_eqPYK_* = 6500 mM [12]
GAPDH for *S. solfataricus*	$\frac{V_{mGAPDH}\times \left(\frac{GAP\times NADP\times Phosphate}{K_{mGAPDHGAP}\times K_{mGAPDHNADP}\times K_{mGAPDHPhosphate}}-\frac{BPG\times NADPH}{\left(K_{eqGAPDH}\times K_{mGAPDHGAP}\times K_{mGAPDHNADP}\right)}\right)}{\left(\left(1+\frac{GAP}{K_{mGAPDHGAP}}\right)\times \left(1+\frac{Pii}{K_{mGAPDHP\mathrm{hosphate}}}\right)+\frac{BPG}{K_{mGAPDHBPG}}\right)\times \left(1+\frac{NADP}{K_{mGAPDHNADP}}+\frac{NADPH}{K_{mGAPDHNADPH}}\right)}$	*V_mGAPDHr_* = 66 mM [6]
		*K_mGAPDHGAP_* = 3.10 mM [35]
		*K_mGAPDHNADP_* = 0.20 mM [35]
		*K_mGAPDHNADPH_* = 0.094 mM [35]
		*K_mGAPDHBPG_* = 0.089 mM [35]
		*K_mGAPDHhospahte_* = 108.52 mM [35]
PGK for *S. solfataricus*	$\frac{V_{m\mathrm{rPGK}}\times \left(\frac{-ATP\times 3PG}{K_{mPGKATP}\times K_{mPGK3PG}}+\frac{K_{\mathrm{eq}PGK}\times ADP\times BPG}{K_{mPGKATP}\times K_{mPGK3PG}}\right)}{\left(1+\frac{ADP}{K_{iADP}}\right)\times \left(1+\frac{3PG}{K_{mPGK3PG}}\times \left(1+\frac{ATP}{K_{mPGKATP}}\right)+\frac{BPG}{K_{mPGKBPG}}+\left(1+\frac{ADP}{K_{mPGKADP}}\right)\right)}$	*VmrPGK* = 73 mM/min [34]
		*KmPGKADP* = 0.374 mM [35]
		*KmPGKATP* = 9.303 mM [35]
		*KmPGKBPG* = 0.008 mM [35]
		*KmPGK3PG* = 0.567 mM [35]
		*KiADP* = 1.14 mM [35]
		*KeqPGK* = 3793 mM ^3^
GAPN for *S. solfataricus*	$\frac{\frac{V_{mGAPN}\times GAP\times NADP}{K_{mGAPNGAP}\times K_{mGAPNNADP}}}{\left(1+\frac{GAP}{K_{mGAPNGAP}}\right)\times \left(1+\frac{NADP}{K_{mGAPNNADP}}\right)}$	*V_mGAPN_* = 20 mM/min [34]
		*K_mGAP_* = 0.02 mM [34] ^4^
		*K_mNADP_* = 0.09 mM [34] ^4^
BPG degradation	KdBPG×BPG	*KdBPG* = 1.058 min^−1^ [7]

^1^ As we could find the forward reaction (i.e., in the direction of 3PG) V_max_ of the *S. cerevisiae* cell extract measured under in vivo conditions, we used the reverse V_max_ for this reaction, therefore we changed the rate law accordingly; ^2^ V_mENO_ was not measured, so we calculated the average differences of measured V_max_ of the enzymes between *S. cerevisiae* and *S. solfataricus* [33,34] and then obtained V_mENO_ according this average difference; ^3^ In Section 4.2.1, we detail how we calculated *K*_eq_; ^4^ Glucose-1-phosphate can influence GAPN activity and also the K_mGAP_ and K_mNADP_ for GAPN. In our model we used the parameters measured [34] at 10 µM glucose-1-phosphate. In order to keep our model simple, we did not include glucose-1-phosphate explicitly.

## Supplementary Materials

Supplementary files in the folder contain the models named “copasi” and a word file called “Thermodynamics”, they are available online at www.mdpi.com/1422-0067/18/4/876/s1. The models are available at JWS-online: https://jjj.bio.vu.nl/.
