# Supplementary material for: The Peculiar Glycolytic Pathway in Hyperthermophylic Archaea: Understanding Its Whims by Experimentation In Silico
Source: Int J Mol Sci. 2017 Apr 20;18(4):876. doi: 10.3390/ijms18040876 (PMC5412457; doi:10.3390/ijms18040876)
Supplement: Supplementary file 1 [file ijms-18-00876-s001.zip › Thermodynamics.pdf]

Thermodynamics

1. For the calculation of equilibrium constant (Keq) for PGK in *S. solatarius*, we used the following formula (Formula 1).

$$\mathrm{Keq_{PGK}} = \frac{[V_{mfPGK}][K_{PGKATP}][K_{PGK3PG}]}{[V_{mrPGK}][K_{PGKADP}][K_{PGKBPG}]} = \frac{\frac{37.96U}{mg}\times 9.309mM\times 0.567mM}{\frac{17.212U}{mg}\times 0.374mM\times 0.0082mM} = 3793 \quad (1)$$

2. For the calculation of Keq for GAPDH in *S. solatarius*, we used the following formula (Formula 1).

$$\mathrm{Keq_{GAPDH}} = \frac{[V_{mfGAPDH}][K_{GAPDHNADPH}][K_{GAPDHBPG}]}{[V_{mrGAPDH}][K_{GAPDHNADP}][K_{GAPDHGAP}][K_{GAPDHPi}]} = \frac{\frac{35.551U}{mg}\times 0.09445mM\times 0.0895mM}{\frac{23.6115U}{mg}\times 0.204mM\times 3.1091mM\times 108.32mM} = 1.85\times 10^{-4} mM^{-1} \quad (2)$$

3. For Keq of PGK in *S. cerevisiae* we used the one in the model the value was 3200.

4. While for Keq of GAPDH in *S.cerevisiae*, we calculated it again according to the following formula (3).

$$Keq = \frac{keq}{K_{Pi}} = \frac{0.0054}{0.0015M} = 3.60M^{-1} \quad (3)$$

Where keq 0.0054 is the value in the model, while K_{Pi} is 0.0015M.

5. In order to investigate the influence of phosphate for GAPDH, we named standard Gibbs free energy without phosphate as $\Delta G^{0\prime}$ and calculated using the following formula (4).

$$\Delta G^{0'} = -RT\ln(Keq) \quad (4)$$

Where in the formula R is gas constant and its value is $8.3kJ^{-1}mol^{-1}$, T is temperature in Kelvin, for calculation $\Delta G^{0\prime}$ of *S. solatarius* T value is 347 K; for calculation $\Delta G_{0}''$ of *S. cerevisiae* T value is 307 K.

The following are the caculations.

$$\Delta G^{0\prime}(S.s) = -RT\ln(Keq) = -\frac{8.314J}{mol\bullet K}\times 343K\ln\left(\frac{0.18}{M}\right) = \frac{4.9kJ}{mol}$$

$$\Delta G^{0\prime}(S.c) = -RT\ln(Keq) = -\frac{8.314J}{mol\bullet K}\times 303K\ln\left(\frac{3.6}{M}\right) = -\frac{3.2kJ}{mol}$$

6. The standard Gibbs energy change for GAPDH with influence of phosphate we named as $\Delta G^{0\prime\prime}$, we used the following formula (5) to calculate $\Delta G^{0\prime\prime}$.

$$\Delta G^{0''} = \Delta G^{0'} + RT\ln\frac{1}{Pi} \tag{5}$$

Where Pi is 10mM,100mM separately.

$$\Delta G^{0''}(S.s: Pi = 1mM) = \Delta G_0{}' + RT\ln\frac{1}{[Pi]} = \frac{4.9kJ}{mol} + \frac{8.314J}{mol\bullet K}\times 343K\ln\left(\frac{1}{0.001}\right) = \frac{24.4kJ}{mol}$$

$$\Delta G^{0''}(S.s: Pi = 10mM) = \Delta G_0{}' + RT\ln\frac{1}{[Pi]} = \frac{4.9kJ}{mol} + \frac{8.314J}{mol\bullet K}\times 343K\ln\left(\frac{1}{0.01}\right) = \frac{17.9kJ}{mol}$$

$$\Delta G^{0''}(S.s: Pi = 100mM) = \Delta G_0{}' + RT\ln\frac{1}{[Pi]} = \frac{4.9kJ}{mol} + \frac{8.314J}{mol\bullet K}\times 343K\ln\left(\frac{1}{0.1}\right) = \frac{11.4kJ}{mol}$$

$$\Delta G^{0''}(S.s: Pi = 1M) = \Delta G_0{}' + RT\ln\frac{1}{[Pi]} = \frac{4.9kJ}{mol} + \frac{8.314J}{mol\bullet K}\times 343K\ln\left(\frac{1}{1}\right) = \frac{4.9kJ}{mol}$$

$$\Delta G^{0''}(S.c: Pi = 1mM) = \Delta G_0{}' + RT\ln\frac{1}{[Pi]} = \frac{-3.2kJ}{mol} + \frac{8.314J}{mol\bullet K}\times 303K\ln\left(\frac{1}{0.001}\right) = \frac{14.2kJ}{mol}$$

$$\Delta G^{0''}(S.c: Pi = 10mM) = \Delta G_0{}' + RT\ln\frac{1}{[Pi]} = \frac{-3.2kJ}{mol} + \frac{8.314J}{mol\bullet K}\times 303K\ln\left(\frac{1}{0.01}\right) = \frac{8.4kJ}{mol}$$

$$\Delta G^{0''}(S.c: Pi = 100mM) = \Delta G_0{}' + RT\ln\frac{1}{[Pi]} = \frac{-3.2kJ}{mol} + \frac{8.314J}{mol\bullet K}\times 343K\ln\left(\frac{1}{0.1}\right) = \frac{2.6kJ}{mol}$$

$$\Delta G^{0''}(S.c: Pi = 1M) = \Delta G_0{}' + RT\ln\frac{1}{[Pi]} = \frac{-3.2kJ}{mol} + \frac{8.314J}{mol\bullet K}\times 303K\ln\left(\frac{1}{1}\right) = \frac{-3.2kJ}{mol}$$

7. As phosphate has no influence on the standard Gibbs energy change for PGK, we used the following formula to calculate $\Delta G^{0''}$ for PGK

$$\Delta G^{0''} = -RT\ln(Keq) \tag{6}$$

$$\Delta G^{0''}(S.s) = -RT\ln(Keq) = -\frac{8.314J}{mol\bullet K}\times 343K\ln(3793) = -\frac{23.5kJ}{mol}$$

$$\Delta G^{0''}(S.c) = -RT\ln(Keq) = -\frac{8.314J}{mol\bullet K}\times 303K\ln(3200) = -\frac{20.3kJ}{mol}$$

8. While for the calculation of ***ΔG***, we used the following formula (7).

$$\Delta G = \Delta G^{0''} + RT \ln \frac{[P_1][P_2]}{[S_1][S_2]} \tag{7}$$

Where in the formula [P1] and [P2] represent the product concentrations at steady state and [S1] and [S2] represent substrate concentrations at steady state.

First we calculated the Gibbs energy change for GAPDH in *S. solfataricus* using the steady state substrates and products concentration values calculated by the model named 'S. solfataricus hy1'.

$$\Delta G(S.s : Pi = 10mM) = \Delta G^{0''} + RT \ln \frac{[BPG][NADPH]}{[GAP][NADP]} = \frac{17.9kJ}{mol} + \frac{8.314J}{mol \bullet K} \times 343K \times \ln \frac{[6.93 \times 10^{-6}][0.39]}{[0.15][1.2]} = -\frac{13.8kJ}{mol}$$

For the Gibbs energy change for PGK in *S. solatarícus* using the steady state substrates and products concentration values calculated by the model named 'S. solfataricus hy1 '.

$$\Delta G(S.s : Pi = 10mM) = \Delta G^{0''} + RT \ln \frac{[3PG][ATP]}{[BPG][ADP]} = -\frac{23.5kJ}{mol} + \frac{8.314J}{mol \bullet K} \times 343K \times \ln \frac{[0.0052][2.52]}{[6.93 \times 10^{-6}][1.32]} = -\frac{2.8kJ}{mol}$$

Then we calculated the Gibbs energy change for GAPDH in *S. cerevisiae* using the steady state substrates and products concentration values calculated by the model named 'S. cerevisiae hy1'

$$\Delta G\ (S.c : Pi = 10mM) = \Delta G^{0''} + RT \ln \frac{[BPG][NADH]}{[GAP][NAD]} = \frac{8.374kJ}{mol} + \frac{8.314J}{mol \bullet K} \times 303K \times \ln \frac{[0.007][0.39]}{[0.15][1.2]} = -\frac{2.2kJ}{mol}$$

Then we calculated the Gibbs energy change for PGK in *S. cerevisiae* using the steady state substrates and products concentration values calculated by the model named 'S. cerevisiae hy1'

$$\Delta G\ (S.s : Pi = 10mM) = \Delta G^{0''} + RT \ln \frac{[3PG][ATP]}{[BPG][ADP]} = -\frac{20.31kJ}{mol} + \frac{8.314J}{mol \bullet K} \times 303K \times \ln \frac{[9.82][2.52]}{[0.007][1.32]} = -\frac{0.42kJ}{mol}$$
